# Emotional, physiological, biochemical, and behavioral responses to acute stress and uncertainty in military personnel

**DOI:** 10.1371/journal.pone.0312443

**Published:** 2024-11-21

**Authors:** Julie A. Cantelon, Ester Navarro, Tad T. Brunyé, Marianna D. Eddy, Nathan Ward, Ida Pantoja-Feliciano, Jordan Whitman, Manob Jyoti Saikia, Grace E. Giles

**Affiliations:** 1 U.S. Army DEVCOM Soldier Center, Natick, Massachusetts, United States of America; 2 Center for Applied Brain and Cognitive Sciences, Tufts University, Medford, Massachusetts, United States of America; Murdoch University, AUSTRALIA

## Abstract

Stress carries diverse implications for perceptual, cognitive, and affective functions. One population particularly susceptible to acute stress-induced cognitive changes are individuals with high-stress jobs (e.g., military personnel). These individuals are often tasked with maintaining peak cognitive performance, including memory, spatial navigation, and decision-making under threatening and uncertain conditions. Previous research has separately examined decision-making under conditions of stress or uncertainty (i.e., ambiguous discrimination between friends and foes). However, questions remain about how operationally relevant stress impacts memory encoding and recall, or spatial learning, as well as how uncertainty may impact decision-making during stress. To address this gap, we examined the influence of a military-relevant emotional stressor on a series of cognitive tasks including recognition memory task (RMT), spatial orienting task (SOT), and shoot/don’t shoot decision making (DMT). To examine the effects of uncertainty and stress we varied the stimulus clarity in the DMT. We utilized threat of shock (TOS) as a high-stakes outcome for decision errors. TOS increased sympathetic arousal but did not affect subjective emotional or HPA responses. TOS influenced decision times and confidence ratings in the DMT, but not response sensitivity or response bias. DMT performance varied by stimulus clarity (uncertainty) but did not differ between stress conditions. TOS did not influence recognition memory or spatial orienting. In sum, high levels of stress and uncertainty characterize military operations, yet stress experienced in military contexts can be difficult to induce in laboratory settings. We discuss several avenues for future research, including methodological considerations to better assess the magnitude and specificity of emotional stress-induction techniques in Soldiers.

## Introduction

Stress occurs when an individual perceives that the demands of a situation (environmental or social) exceeds their adaptive abilities [1]. Situations that elicit stress are often novel, uncontrollable, unpredictable, or threatening [2, 3]. Military and law enforcement personnel are frequently challenged with maintaining cognitive performance under highly demanding and uncertain circumstances. Peak performance in such stressful conditions requires heightened cognitive control to maintain focus and execute behaviors that align with objectives. Research shows that moderate to high stress levels can adversely influence performance on tasks requiring executive processes, memory retrieval, and prefrontal cortical function [4–9].

Importantly, although measuring cognitive performance in applied settings (i.e. field studies) may provide greater ecological validity than in the laboratory, field studies involve substantial variability, as well as methodological and practical limitations [10, 11]. In turn, it is difficult to disentangle effects of stress because cognitive changes are likely multiplied by the ever-changing demands of the mission, as well as operational factors such as sleep loss, fatigue, thermal load, dehydration, resource constraints [12–15]. Thus, research examining how stress influences cognitive outcomes relevant to military operations, like moving, shooting and communicating, under more controlled settings is needed in order to better monitor and predict degradations in real time, or in the future.

### Eliciting Soldier stress in the lab

Various paradigms have been used to elicit acute emotional stress in laboratory settings. These include social-evaluative threat (e.g., Trier Social Stress Test), threat of aversive shock (e.g., finger or torso shock), noxious noise exposure, the cold pressor test, exposure to aversive images or videos (e.g., International Affective Picture System) and demanding cognitive tasks [16–20]. However, most laboratory-grade stress and anxiety inductions are relatively mild (e.g., public speaking, or submerging an arm in cold water) [21, 22] and may fail to reflect the intensity, frequency, and/or duration of stressors experienced by Soldiers during training and operations.

Threat of Shock (TOS) may be one method for eliciting acute and operationally relevant stress in laboratory settings. The electric shock presents a dual threat, constituting a physical threat to comfort and a psychological threat due to its unpredictable nature, leading to anticipatory anxiety. Responses to this stress can be assessed subjectively via questionnaire measures or objectively via measurement of sympathetic-adrenomedullary (SAM) and the hypothalamic–pituitary–adrenal (HPA) axis responses. SAM response occurs rapidly and can be measured through skin conductance, pupil dilation, heart rate or free salivary α-amylase 10 to 12 min after stress cessation [23]. In contrast, the HPA axis response is slower and typically peaks about 30 to 60 min following the stressful event, when measured via salivary cortisol [21, 24]. For example, moderate-intensity electric shock is associated with increased subjective anxiety ratings, hypervigilance to threat, as well as sustained salivary cortisol and α-amylase responses, higher heart rate, and lower heart rate variability, compared to either no shock or vibrate conditions [16, 17, 25–31]. In a previous meta-analytic review of laboratory stress induction techniques, TOS was considered to be one of the more intense physical and psychological stressors [16]. Additionally, performance-contingent manipulations of TOS (i.e., the anticipation of aversive shock in the case of performance failure) may better represent stressors experienced in high-stress domains (e.g., military or law enforcement personnel). Previous work shows that this can emulate the anxiety experienced in response to anticipation of adverse consequences of error making, like the return of hostile fire [32, 33].

### Threat of shock and performance

An emotional stress response to threat, such as response to TOS, can cause decisive behavior to occur before a cognitive evaluation [34] and can negatively impact decision making [35]. Indeed, acute stress enhances defensive responses aimed at safeguarding the organism from threats (such as an increased startle reflex or heightened environmental vigilance), while impairing higher-level cognitive processes that are less vital for immediate harm avoidance [36–40]. For instance, attentional bias and hypervigilance to threat, such as that induced by TOS, can lead to competition for attentional and sensory-perceptual resources [41–45] resulting in impaired performance on tasks involving working memory [46–49] and cognitive flexibility [50].

In potentially dangerous environments, stress can promote focus and sustain attention to potential threat, which may be adaptive for survival. This serves to improve response inhibition, reducing the likelihood of habitual or impulsive responding when threat is present, promoting harm avoidance [45]. However, impairments to response inhibition may also help promote harm avoidance. When in a threatening context, it may be adaptive to adopt a liberal decision criterion and respond as though all stimuli pose a threat, as missing a threat could have dire consequences [51–53]. In line with this, previous work has shown that threat of shock leads to shifts in response criterion, where participants correctly identify enemy targets (hits) at the expense of increased false alarms for friendly targets on shoot/don’t shoot tasks [17, 29, 39]. Thus, acute stress elicited from TOS may impair cognitive control and impact discrimination of friends versus foes necessary for shoot or don’t shoot decisions.

In contrast, the extent to which threat of shock affects other processes essential to Soldier operations, such as spatial learning, or memory encoding and recall remain relatively unexplored. Research shows that the impact of stress on memory depends on the context and memory modality. For instance, working memory may be impaired, whereas long-term memory may be enhanced by TOS (see [45] for review). Indeed, research shows that spatial working memory is disrupted by TOS regardless of task difficulty [47–49, 54, 55], yet the extent to which long-term memory or spatial memory are affected by TOS is less understood. The timing of the stressor seems to drive the effects on long-term memory, where stress during encoding may facilitate but stress during recall may impair memory performance [45, 56]. Soldiers are required to engage memory processes during operations, like remembering persons or objects from a “Be On The Lookout” (BOLO) list, or quickly navigating through a previously learned environment. Learning and recall may occur under safe or threatening conditions. Yet, previous TOS research has primarily focused on threat-encoding/threat-retrieval conditions [57, 58], with limited work examining effects of TOS when information is learned under safe conditions and retrieved under threat.

### Uncertainty and performance

An additional factor of relevance in real-world high-stakes contexts is uncertainty. Soldiers and law enforcement personnel are continually called upon to quickly interpret ambiguous information in uncertain and threatening environments. One of the most critical decisions is threat versus non-threat determinations. In some cases, threats versus non-threats can be easily discriminated and have been the focus of much of the prior work on shoot or don’t shoot decision errors [59–64]. However, oftentimes decisions are made with some degree of uncertainty due to limited or insufficient information required to predict outcomes of behavior [65]. Previous research suggests that unpredictability is aversive [27, 66, 67] and elicits heightened responses to uncertain stimuli in brain regions associated with threat detection [68]. As such, research in the affective domain has explored how stress impacts decisions about emotional uncertainty (i.e., valence bias), finding that TOS leads to negative interpretations of ambiguous stimuli (surprised faces) [69, 70]. Additionally, extensive laboratory research has focused on perceptual decision-making under uncertain circumstances where factors like insufficient visual information make it challenging to distinguish stimuli (e.g. low image clarity). However, research on uncertainty in perceptual decision making has primarily done so using computer-based tasks whose outcome is, at best, tied merely to small monetary incentives [71–75]. Questions remain about how uncertainty impacts more high stakes decisions, especially during stressful conditions.

Anticipation of aversive states and experiences (e.g., threat of shock) can trigger a cascade of emotional and neuroendocrine stress responses [76]. Such acute emotional stress is associated with increased negative affect, reduced cognitive control, and flexibility, and reduced regulation of negative emotion [77, 78], which have been shown to alter cognitive performance. Furthermore, stress may alter responses to uncertainty in high stakes contexts [79]. In the present study we examine how TOS impacts memory recognition and spatial navigation, and how TOS and stimulus uncertainty influences high stakes decisions.

## Methods

Note that because this study was accomplished immediately upon the post-SARS-CoV-2 reopening of Tufts University, all experimenters and participants followed approved (by safety committees at Tufts University and U.S. Army) safety protocols including procedures for personal protective equipment (e.g., masks, gloves, glasses, gowns, face shields), biospecimen handling, hand sanitizing, social distancing, and equipment and space sanitization.

### Participants & design

This study used a repeated-measures design, with threat (shock, vibrate) as the within-participants factors. Recruitment for this study began January 5^th^, 2021, and ended on August 8^th^, 2021. Data from 79 Soldiers are reported herein (79 men, 0 women). Both males and females were recruited for this study, only males enrolled. Therefore, the participants represented a healthy male Soldier population. Sample characteristics are listed in Table 1. Written informed consent was obtained, and the U.S. Army Combat Capabilities Development Command Armaments Center Institutional Review Board, Tufts University Institutional Review Board, and the Army Human Research Protections Office approved all procedures.

**Table 1:** Sample characteristics.

	*n*	%	Mean	Median	SD	Minimum	Maximum
Age (years)			23.1		3.8	18	35
Sex			2.6	2	2.2	0	11
Male	79	100%					
Female	0	0%					
Years in Military			2.6	2	2.2	0	11
Number of Deployments			0.3	0	0.5	0	2
Race and Ethnicity							
White	59	74.7%					
Black	2	2.5%					
Hispanic	13	16.5%					
Asian	2	2.5%					
Other	3	3.8%					
Highest Education Level							
Some High School	1	1.3%					
High School/GED	43	54.4%					
Some College	25	31.6%					
Associate’s degree	5	6.3%					
Bachelor’s degree	5	6.3%					
Graduate degree	0	0%					

#### Decision Making under Uncertainty and Stress task

**Fig 1:**
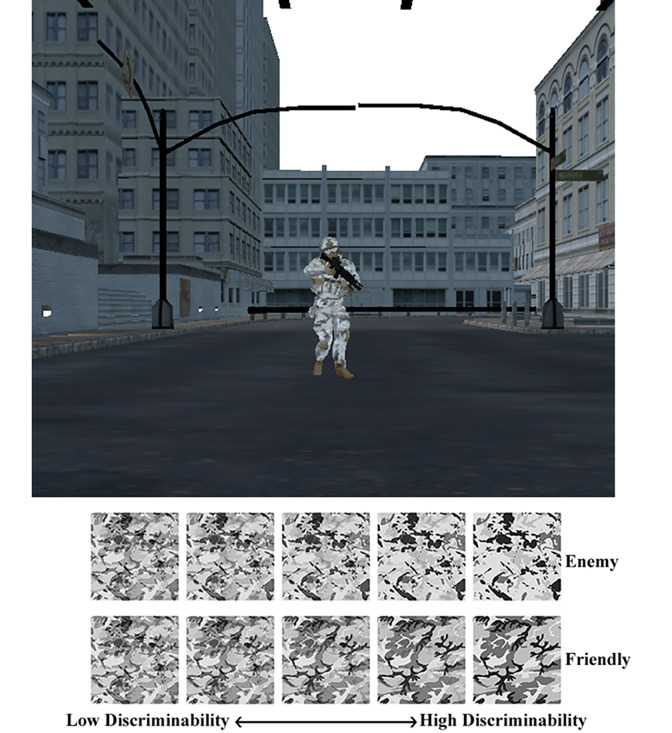
Example of camouflage patterns for friendly and enemy targets ranging in clarity from low discriminability (51% clarity) to high discriminability (100% clarity).

The Decision Making under Uncertainty and Stress (DeMUS) task was designed to elicit uncertainty and examine perceptual decision-making [72, 73, 80, 81]. Complete details of scenario development and task methodology are described elsewhere (see [82]). In this task, participants were asked to mark remembered objects from a BOLO list, orient toward waypoints in a virtual environment, and distinguish friendly and enemy targets based upon the camouflage patterns worn by virtual avatar. They also rated the confidence in their decisions on a scale from 1 (*least confident*) to 5 (*most confident*). To introduce uncertainty, friendly and enemy camouflage patterns were layered atop one another using Photoshop. We then varied the top layer opacity so even if the patterns may be indistinguishable at a 51% opacity (i.e., lowest clarity), there was always an objectively correct answer (e.g., enemy pattern- 51% enemy and 49% friendly) (see Fig 1 for example). For complete details of development and methodology of the DeMUS tasks described below, see [82].

1. *Criterial Learning Tasks (CLT)*. During this task, participants learned the stimuli to be used in the DeMUS tasks to a pre-specified criterion (i.e. learned targets for the recognition memory task (RMT), a map of the virtual environment for the spatial orienting task (SOT) and friendly and enemy camouflage patterns for the shoot/don’t shoot decision making task (DMT)). For the DMT CLT, participants were presented with the two canonical (100%) versions of the friendly and enemy camouflage patterns; each of the four versions of the shoot/don’t shoot task used a unique pairing of camouflage patterns (as described in [82]). During the test, the low uncertainty (always 100% clarity) and moderate uncertainty (typically 60–70% clarity) versions of the patterns were presented, one at a time on the computer monitor, in random order. Participants were never exposed to the high uncertainty stimulus (51% clarity) versions during the criterial learning test. This criterial learning phase ensured that all participants had learned all DeMUS task-related stimuli to at least .8 accuracy.
2. *Recognition Memory Task (RMT)*. For this task, participants were dropped at several locations around the virtual environment and asked to look for previously learned targets (e.g., graffiti markings, people, vehicles). For Soldiers, this would be similar to recognizing targets on a BOLO list. Dependent measures included discriminability (d’, ability to discern targets from lures).
3. *Spatial Orienting Task (SOT)*. For this task, participants were dropped in a series of locations around the virtual environment and asked to orient towards previously learned waypoints. For example, a participant might be placed next to the Library and asked to point in the general direction of the Drug Store. Dependent measures included distance error (estimated from actual distance to actual distance), distance confidence (confidence in distance estimate), direction error (ratio of estimated from actual direction to 180°), and direction confidence (confidence in direction estimate).
4. *Shoot/Don’t-Shoot Decision Making Task (DMT)*. For this task, participants are asked to discriminate between friendly and enemy targets based upon the camouflage pattern worn by virtual avatars. An animated soldier avatar would walk towards the participants, and the participant decided whether to shoot the target (enemy) or let it pass (friendly). Dependent measures included discriminability (d’, ability to discern enemies from friends), decision distance (mean of the target distance when the participant made a decision), and confidence (ranging from 1–5).

#### Threat of shock

To induce stress, electric shock was delivered using a StressX PRO Belt (StressVest, Winnipeg, Canada) worn around the torso. This belt can deliver up to 4500 volts at less than 1mA over a maximum discharge duration of 150ms (maximum joules 0.092 or 92mJ). The belt used 5 amperage-modulating intensity settings ranging from lowest (1) to highest (5) shock intensity. The device was triggered wirelessly by a base station connected to the virtual reality computer.

Participants first performed a shock calibration since they likely differed in their tolerance of varying shock intensity levels. During the calibration, participants were asked to don the StressX PRO shock belt around the torso and self-administer shocks (using a provided button), starting at the lowest intensity (1) and stopping when they were no longer comfortable increasing the intensity. Participants then removed the belt and experimenter recorded the highest shock intensity achieved by the participant (e.g., 3). In accordance with our approved safety protocol, one level below the highest intensity value achieved (e.g., 2) was used during the DeMUS task. If a participant did not try above level 1, level 1 was used in the DeMUS task. In the shock condition, participants received a shock upon making an incorrect response across all trials on the RMT, SOT and DMT tasks (e.g., incorrect identification of a BOLO item, orientation towards a waypoint, miss or false alarm). In the no shock condition, participants received a vibration from the belt upon making an error. Thus, task-related feedback was maintained in both conditions.

#### Zephyr Bioharness

The induction of stress or the presence of threat increases heart rate [83]. Continuous heart rate monitoring was collected using a Zephyr BioModule device and chest strap (Medtronic, Boulder, CO) as a manipulation check to determine if the threat of shock procedure reliably induced sympathetic arousal in participants [47, 48]. Heart rate was collected during a 5-minute seated baseline period, prior to beginning the DeMUS task (while the experimenter set up the testing computers) and during shock and vibrate conditions. Heart rate was measured in beats per minute at 1Hz (derived from an 18Hz R-R interval extracted from contiguous 250ms blocks of electrocardiogram/ECG data) and averaged across baseline, RMT, SOT and SMT phases of the DeMUS task for shock and vibrate conditions. As each phase of the DeMUS scenario contained the possibility of shock, we examined how heart rate varied over time as a function of time exposed to threat of shock. Heart rate data from 52 participants are reported herein due to technical errors with Bioharness equipment.

#### Questionnaires

*State-Trait Anxiety Inventory-State (STAI-S)*. The STAI-S is a 10-item subscale that measures state anxiety: how participants feel at that particular moment, ranging from “not at all” (1) to “very much so” (4) [84]. Scores range from 20 to 80, with higher scores indicating higher levels of anxiety. Participants completed the STAI-S pre and 0 minutes post the virtual DeMUS scenario.

*Positive and Negative Affect Schedule (PANAS)*. The PANAS is a 20-item scale, with 10 items measuring positive and 10 measuring negative affect [85]. Items are rated on a 5-point Likert scale (1- “very slightly or not at all”; 5- “extremely”). Scores range from 10–50 with higher scores indicating higher levels of affect. Participants completed the PANAS pre and 0 minutes post the virtual DeMUS scenario.

### Procedure

Participants visited the laboratory on two separate occasions, separated by 24 hours. Both sessions took place between approximately 7:00 am and 11:00 am. Upon arrival, participants were seated in a private testing room. They completed pre-task questionnaires on a computer monitor (24” at 1920×1080 resolution, with standard keyboard and mouse). These included the STAI, PANAS, the simulator sickness questionnaire (SSQ) [86]. They then provided their first of five saliva samples. All samples were taken using the SalivaBio Oral Swab method (Salimetrics, LLC, Carlsbad, CA), which involves placing a swab under the tongue for 2 minutes in order to collect approximately 2 mL saliva volume, the placing the swab into a storage tube, and storing the sample in a -20° C freezer until analysis. To ensure quality of saliva samples, participants were instructed to abstain from eating a major meal within 60 minutes of their study sessions and to abstain from tobacco-and nicotine-containing products on the day(s) of their study sessions. Next, participants completed the shock calibration (described earlier) and then began the CLT to confirm adequate memory (minimum .8 accuracy) for BOLO targets in the RMT, map waypoints in the SOT and camouflage patterns in the DMT.

**Fig 2:**
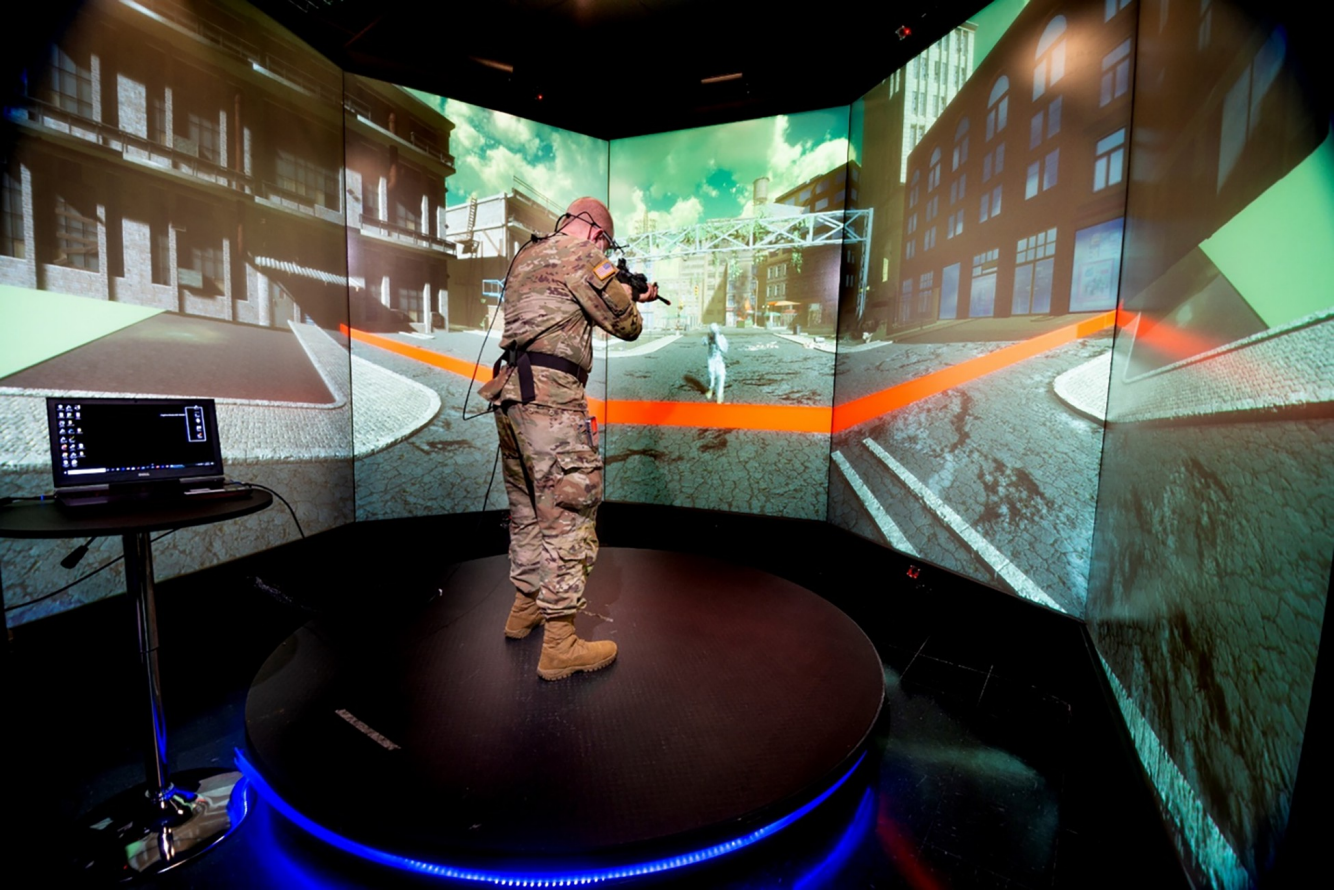
Example of the virtual reality system used to execute Decision Making under Uncertainty and Stress task.

Next, participants were escorted to the virtual reality system. They then donned the Bioharness chest strap and were given instructions on how to use the rifle and attached controller (button press, thumb joystick). They then stepped onto a rumble platform, which was in the center of the VR system, surrounded by five display panels. See Fig 2 for example of virtual scenario.

To gather baseline physiological data, we had participant sit quietly in a chair for ten minutes. Lastly, the StressX PRO Belt was donned and set to the appropriate condition, either shock or vibrate, and to the participant’s predetermined shock intensity level (as described above). Shock condition order was counterbalanced across participants.

Written and verbal instructions were provided, and participants practiced 2–3 trials of the DeMUS tasks, using environments and stimuli that were not previously learned (i.e., one of the other two task versions of the task). Shock was not administered during practice. Once participants were familiar with each DeMUS task, we started the experimental session. One of the four developed virtual scenarios was randomly chosen without replacement for use in each experimental session (shock, vibrate). The DeMUS task began with 10 trials of the RMT and SOT, which took about 20 minutes and then completed 15 trials of the DMT, which took about 10 minutes. The DeMUS task took about 35 minutes to complete from start to finish. Upon completion, the experimenter removed the shock belt and Bioharness and escorted the participant back to the private testing room.

**Fig 3:**
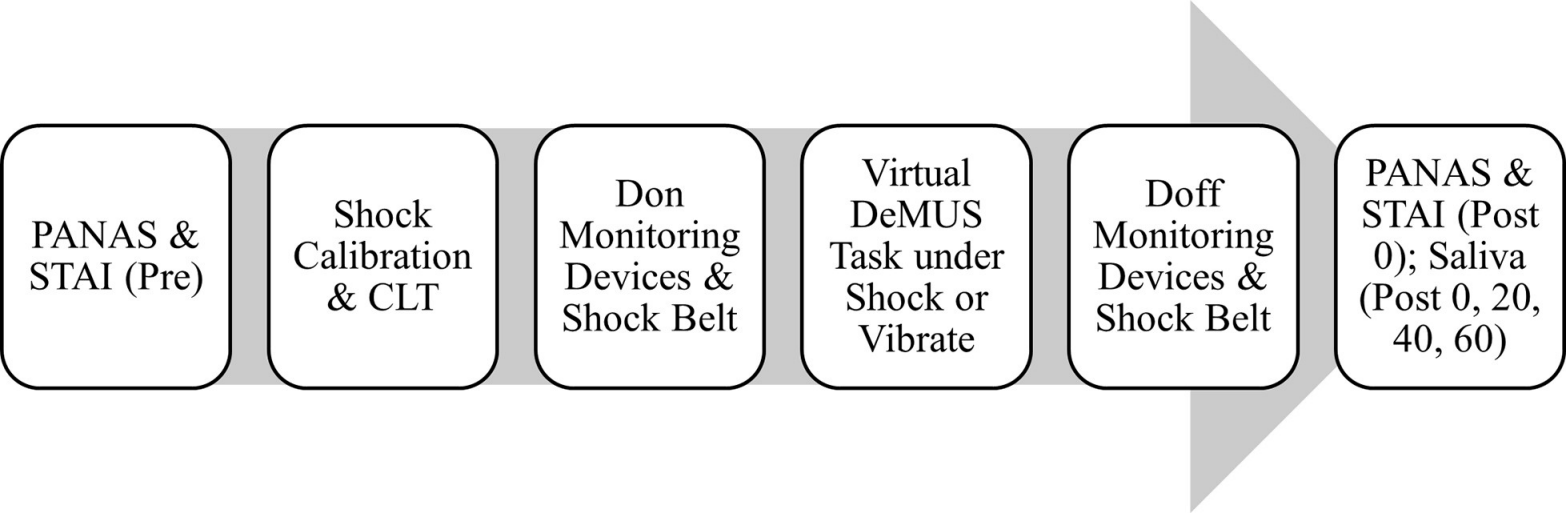
Schematic of experimental sessions. Participants first completed questionnaires, provided a baseline saliva sample and completed shock calibration, followed by the Criterial Leaning Task (CLT). They then donned physiological monitoring devices and the shock belt and completed the Decision Making under Uncertainty and Stress (DeMUS) task. Following the DeMUS task they completed questionnaires and provided a series of saliva samples.

Participants then completed the STAI, PANAS immediately (0) and saliva samples 0, 20, 40, and 60 minutes after completing the DeMUS task. After providing their final saliva sample, participants were thanked for their participation and released for the day. See Fig 3 for a schematic of the experimental sessions.

### Statistical methods

Behavioral performance on the RMT included sensitivity (d’) of object marking, on the SOT included absolute angular error of pointing estimates, response times, confidence ratings, and distance estimation error, and on the DMT included sensitivity (d’) of decision making, response bias (criterion (*c*)), decision distance, and confidence ratings. To explore the effects of stimulus uncertainty, we compared a subset of camouflage patterns, including the 65% and 100% clarity patterns (see [82] for additional details). Sensitivity (*d’*) was then calculated using the following formula: *d’* = *z*(hits) − *z*(false alarms), and response bias or (criterion (*c*)) was calculated as *c* = −0.5 (*z*(hits) + *z*(false alarms)). A value of *d′* = 3 is close to perfect performance; a value of *d′* = 0 is chance (“guessing”) performance. For criterion, negative values indicated a bias toward responding yes (i.e., shooting). Each measure was determined separately for each participant and each stress (shock, vibrate) and uncertainty condition (low, high). A liberal response bias refers to the tendency to identify camouflage patterns as an enemy more frequently than friendly; a conservative response bias refers to the tendency to identify camo patterns as friendly more frequently than an enemy. Decision distance was calculated for hits (correct shoot decisions).

Physiological responses included heart rate, cortisol and alpha amylase. For heart rate, we calculated mean beats per minute (BPM), which was collected in 10-second increments at 1Hz averaged across baseline, RMT, SOT and DMT phases of the DeMUS scenario for shock and vibrate conditions. Analysis for salivary cortisol and salivary alpha-amylase were done using specific Enzyme-Linked Immunosorbent Assay (ELISA) kits in triplicate. Cortisol data were positively skewed (Fisher’s skewness = 3.42), therefore, we performed a fourth-root transformation X^.25 to reduce skewness (Fisher’s skewness = 1.21), as recommended in previous research [87], to ascertain that data met the requirements of general linear model-based statistics in terms of Gaussian distribution. This method has been shown to be superior in terms of meeting normality and homoscedasticity (indicated by skewness and kurtosis) compared to the log-transformation commonly used on times-series endocrine data [88].

To assess differences in performance due to stress, we conducted a repeated measures analysis of variance (ANOVA) with stress condition (shock, vibrate) and, where appropriate, time (pre, 0 minutes after, 20 minutes after, 40 minutes after, 60 minutes after) as within-participants factors. To assess differences in shoot/don’t shoot performance due to stress and uncertainty, we conducted an omnibus 2 (stress condition: shock, vibrate) x 2 (uncertainty: low, high stimulus clarity) repeated measures ANOVA. When an ANOVA yielded a significant main effect or interaction (*p* < .05), post-hoc tests using the Bonferroni corrections were conducted. Effect sizes are provided for all analyses, using η_p_^2^ for ANOVAs, and Cohen’s *d* for *t*-tests.

All statistical analyses were performed using SPSS 25 and R (R Data Core, 2021). Data were assessed for normality using Shapiro-Wilk tests. When sphericity was violated, Greenhouse-Geisser corrections were used. When an ANOVA yielded a significant main effect or interaction (*p* < .05), post-hoc tests using the Bonferroni corrections were conducted. Effect sizes are provided for all analyses, using ηp^2^ for ANOVAs, and Cohen’s d for t-tests.

## Results

### Emotional and physiological responses

#### PANAS

*Positive and Negative Affect Schedule*. There were no effects of Stress or Time, nor interactions for positive affect (*p*s > .14). There was a main effect of Time for negative affect, *F*(1, 74) = 8.1468, *p* < .006 η_p_^2^ = .10, in which negative affect was higher after both shock and vibrate conditions than before (see Table 2). There were no effects of Stress, nor interactions for negative affect (*p*s > .21).

**Table 2:** Positive and negative affect and state anxiety rating means ± SD before and after shock and vibrate conditions (n = 77).

		Time Relative to Stress	
		Before	After
Positive Affect	Shock	28.8 ± 8.6	29.2 ± 8.4
	Vibrate	28.1 ± 8.8	28.8 ± 8.8
Negative Affect	Shock	11.4 ± 3.0^a^	13.1 ± 4.8^b^
	Vibrate	11.9 ± 3.5^a^	12.5 ± 4.6^b^
State Anxiety	Shock	31.2 ± 8.4^a^	33.0 ± 9.1^b^
	Vibrate	31.2 ± 9.3a	32.0 ± 8.9^b^

Means denoted by different lower-case letters indicate significant differences between time points (*p* < 0.05).

#### STAI-S

There was a main effect of Time, *F*(1, 76) = 10.131, *p* < .002, η_p_^2^ = .12, in which state anxiety was higher after both shock and vibrate conditions than before (see Table 2). There were no effects of Stress nor Stress by Time interactions (*p*s > .29).

#### Salivary cortisol

There was a main effect of Time, *F*(4, 284) = 90.704, *p* < .001, ηp^2^ = .561 for salivary cortisol levels. Bonferroni post-hoc analyses revealed that cortisol levels were lower at all time points after than before both shock and vibrate conditions (*p*s < .01) (see Table 3). There were no effects of Stress nor Stress by Time interactions (*p*s >.47).

**Table 3:** Salivary cortisol concentration (µg/dL) and alpha amylase (µ/mL) means ± SEM sampled before and every 20 minutes after shock and vibrate conditions (n = 77).

		Time Relative to Stress				
		Before	0 Min After	20 Min After	40 Min After	60 Min After
Cortisol	Shock	0.78 ± 0.01^a^	0.69 ± 0.01^b^	0.68 ± 0.01^b^	0.67 ± 0.01^b^	0.65 ± 0.01^b^
	Vibrate	0.79 ± 0.01^a^	0.68 ± 0.01^b^	0.69 ± 0.01^b^	0.67 ± 0.01^b^	0.66 ± 0.01^b^
Alpha Amylase	Shock	65.5 ± 0.79^a^	87.1 ± 8.1^b^	83.2 ± 7.0^b^	93.7 ± 9.0^b^	99.5 ± 9.5^b^
	Vibrate	65.5 ± 6.7^a^	87.1 ± 7.1^b^	95.2 ± 8.7^b^	98.9 ± 9.5^b^	97.6 ± 9.4^b^

Means denoted by different lower-case letters indicate significant differences between time points (*p* < 0.05).

#### Salivary alpha-amylase

There was a main effect of Time, *F*(4, 300) = 14.175, *p* < .001, ηp^2^ = .159, in which post-hoc analyses revealed that salivary alpha amylase levels were higher at all time points after than before both shock and vibrate conditions (see Table 3). There were no effects of Stress nor Stress by Time interactions (*p*s > .001).

#### Heart rate

There was a main effect of Time, *F*(1, 52) = 282.577, *p* < .001, ηp^2^ = .23. Post-hoc comparisons revealed that average heart rate (BPM) was higher during DMT than RMT and SOT, and lower during the seated baseline than DMT, RMT and SOT. In a Stress by Time interaction, *F*(2, 104) = 14.449, *p* < .001, ηp^2^ = .004, average heart rate was higher under shock than vibrate during the DMT, *t*(52) = 2.19, *p* = .033, *d* = .033, but no differences between conditions were found during RMT, SOT or the seated baseline (*p*s > .06), see Table 4.

**Table 4:** Heart rate (bpm) means ± SD sampled during a 5-minute seated baseline period before, and during the recognition memory task (RMT), spatial orienting task (SOT), and shoot/don’t-shoot decision making task (DMT) (n = 52).

	Time Relative to Stress		
	Seated Baseline	RMT and SOT	DMT
Shock	74.4 ± 19.3^a^	87.8 ± 22.1^b^	89.8 ± 22.7^bA^
Vibrate	71.5 ± 10.0^a^	82.2 ± 11.2^b^	83.3 ± 11.69^bB^

Means denoted by different lower-case letters indicate significant differences between time points (*p* < 0.05). Means denoted by different upper-case letters indicate significant differences between shock conditions (*p* < 0.05).

### Behavioral performance

#### Effects of shock

For the RMT, no differences were found for discriminability (d’) (*p* = .113), see Table 5.

**Table 5:** Recognition memory task (RMT), spatial orienting task (SOT), and shoot/don’t-shoot decision making task (DMT) performance by shock condition (n = 79).

		Shock		Vibrate	
Task	Metric	M	SD	M	SD
RMT	Discriminability (d’)	0.7	0.1	1.0	0.1
SOT	Distance Error	763.7	24.4	787.4	28.4
	Direction Error	31.3	1.7	33.8	2.0
	Distance Confidence	2.8	0.1	2.8	0.1
	Direction Confidence	3.2	0.1	3.3	0.1
DMT	Discriminability (d’)	1.4	0.1	1.1	0.1
	Decision Distance	40.5^A^	4.8	38.6^B^	4.4
	Confidence	3.3^A^	0.1	3.8^B^	0.1

Means denoted by different upper-case letters indicate significant differences between shock conditions (*p* < 0.05).

For the SOT, no differences were found for distance error, direction error, distance confidence, or direction confidence (*p’*s > .06). For the DMT, decision distance was greater under shock than vibrate, *t*(77) = 2.369, *p* = .027, *d* = .268 and confidence was lower under shock than vibrate, *t*(77) = 4.66, *p* < .001, *d* = .528. No differences were found for d’ (*p* = .064).

#### Effects of uncertainty

**Fig 4:**
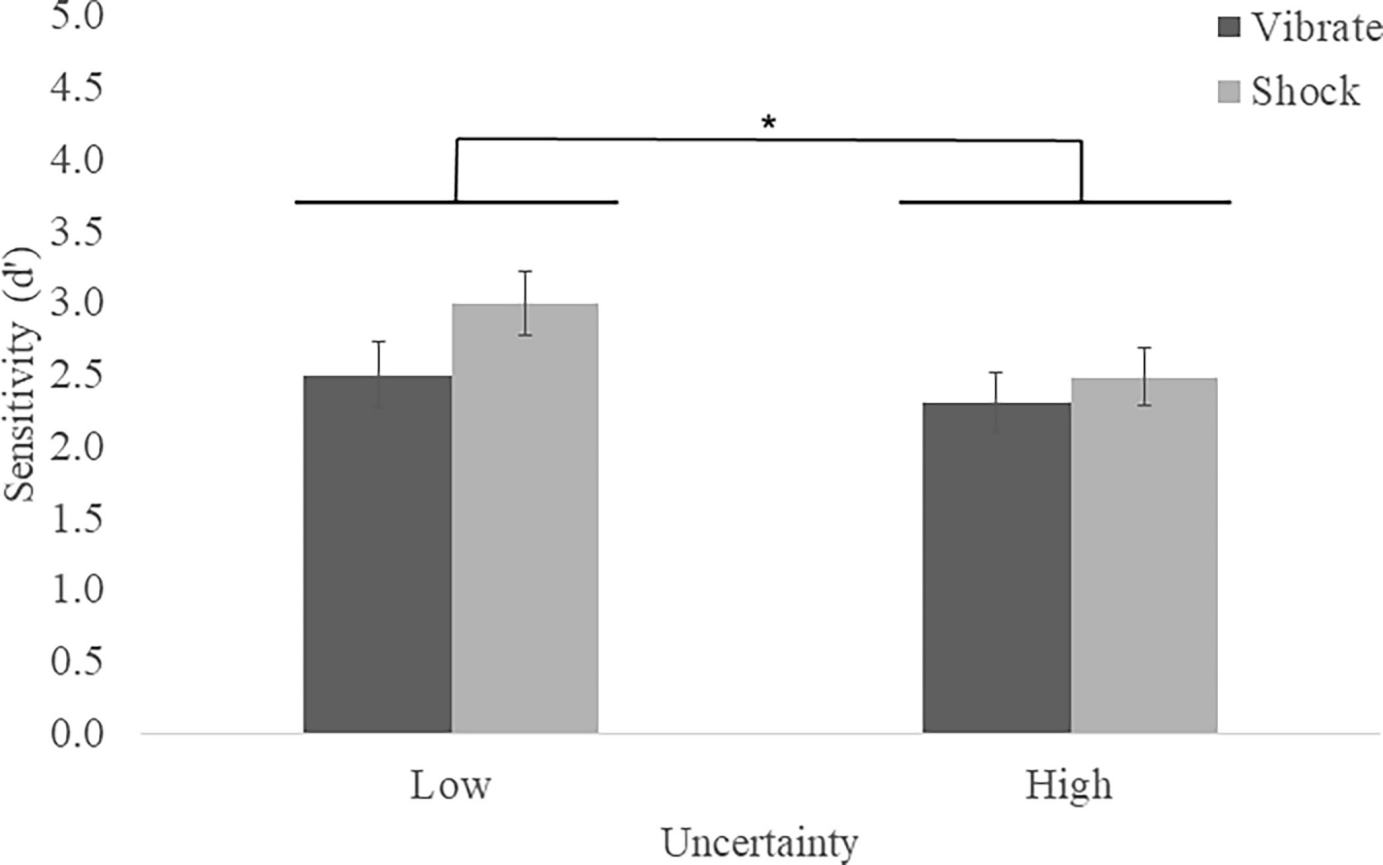
Shoot/don’t shoot sensitivity means (SEM) by stress and uncertainty. Sensitivity did not differ between shock and vibrate conditions (p > .213), but participants were better at distinguishing between enemy and friendly targets in the low compared to high uncertainty condition (*p < .05).

*Sensitivity (d’)*. To explore impacts of uncertainty, we compared friend/foe decisions using a subset of camouflage patterns, including 100% clarity (low uncertainty) and 65% clarity (high uncertainty) stimuli. There was a main effect of Uncertainty, *F*(1, 78) = 4.79, *p* < .032, η_p_^2^ = .06, in which participants were more sensitive to the difference between signal (enemy) and noise (friendly) in the low compared to high uncertainty condition (see Fig 4) There were no effects of Shock nor Shock by Uncertainty interactions (*p*s > .213).

**Fig 5:**
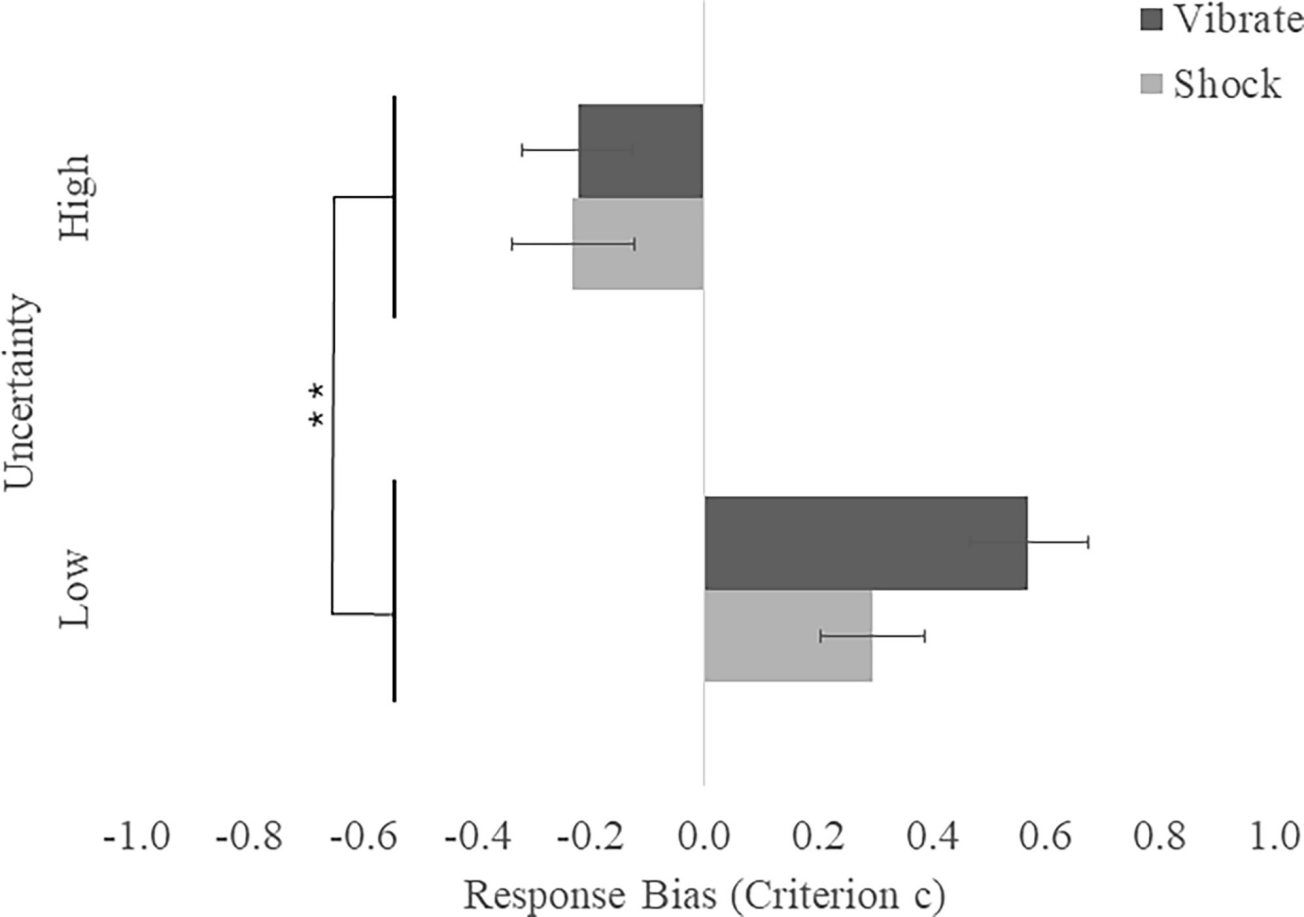
Shoot/don’t shoot response bias means (SEM) by stress and uncertainty. Response bias did not differ between shock and vibrate conditions (*p* > .170), but participants had a more liberal response bias and were more likely identify targets as an enemy than friendly in the high than low uncertainty condition (*p < .001).

*Response bias (Criterion c)*. There was a main effect of Uncertainty, *F*(1, 78) = 32.91, *p* < .001, η_p_^2^ = .30, in which participants were more biased towards responding yes (i.e. shooting) in the high compared to low uncertainty condition (see Fig 5). There were no effects of Stress nor Stress by Uncertainty interactions (*p*s > .170).

*Hits and false alarms*. To further explore changes in response bias between uncertainty conditions we examined hit and false alarm rates. There was a main effect of Uncertainty, *F*(1, 78) = 11.12, p < .001, η_p_^2^ = .14, in which hits were lower in the low uncertainty than high uncertainty condition (see Table 6). This likely contributed to higher response bias observed under high compared to low uncertainty conditions. There were no effects of Stress nor Stress by Uncertainty interactions (*p*s > .084).

**Table 6:** Shoot/don’t shoot hit rate, false alarm rates, and decision distance means ± SD by stress and uncertainty (n = 79).

		Hit Rate		False Alarm Rate	
Stress Condition	Stimulus Uncertainty	M	SD	M	SD
Shock	Low	0.82^1^	0.29	0.12^1^	0.29
	High	0.77^1^	0.27	0.27^1^	0.24
Vibrate	Low	0.64^2^	0.35	0.11^2^	0.23
	High	0.81^2^	0.24	0.29^2^	0.29

Means denoted by different numbers indicate significant differences between uncertainty conditions (*p* < 0.001).

There was a main effect of Uncertainty, *F*(1, 78) = 29.410, *p* < .001, η_p_^2^ = .27, in which false alarms were lower in the low than high uncertainty condition (see Table 6). There were no effects of Stress nor Stress by Uncertainty interactions (*p*s > .64).

**Fig 6:**
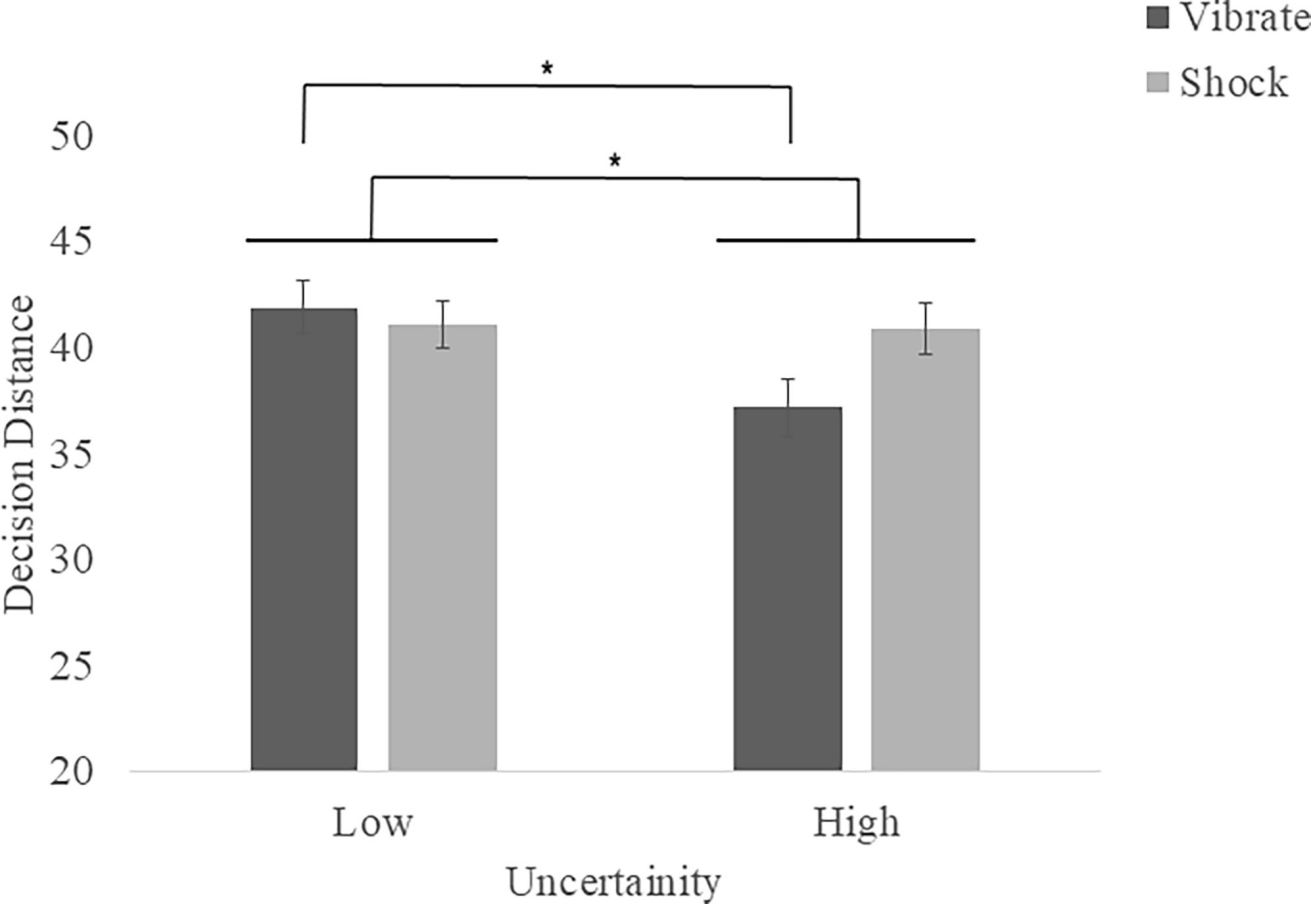
Shoot/don’t shoot response decision distance means (SEM) by stress and uncertainty. Decision distance was lower under high compared to low uncertainty conditions (*p < .05). Specifically, participants were slower to respond (i.e. had a greater decision distance) for low compared to high clarity camouflage patterns in vibrate conditions (*p < .05).

*Decision distance*. When examining decision distance there were no effects of Stress but (*p*s > .19) but a marginally significant main effect of Uncertainty, *F*(1, 60) = 3.28, *p* = .052, η_p_^2^ = .075, and a Stress by Uncertainty interaction, *F*(1, 60) = 5.67, *p* = .020, η_p_^2^ = .086. Bonferroni post-hoc analyses revealed this interaction was driven by lower decision distance (i.e. slower response time) for high compared to low uncertainty patterns in the vibrate condition (*p* = .050) (see Fig 6).

## Discussion

In the present study, we evaluated whether stress induced by threat of shock impacted recognition memory, spatial orienting and shoot/don’t shoot decisions. To examine whether uncertainty impacted decisions made under stress, participants made friend/foe discriminations using high or low clarity camouflage patterns presented on avatars in a virtual environment, while under shock or vibrate conditions. In addition, pre-post measures of positive and negative affect, state anxiety and salivary cortisol and alpha-amylase were collected, and heart rate was collected during both conditions.

### How does threat of shock influence physiological and emotional responses?

We found that threat of shock impacted some physiological responses, but not others. Results showed that heart rate was higher during the shock than vibrate condition during the DMT. Exploration of total shock counts indicated that shock frequency (i.e., error rate) was highest during the DMT phase of the DeMUS task. This elevated heart rate during DMT aligns with previous research demonstrating threat-induced increases in sympathetic autonomic nervous system (ANS) activation [47, 48, 89]. In contrast, salivary cortisol decreased throughout the testing session, consistent with the cortisol awakening response (CAR) in which cortisol level peak early morning and decline throughout the day [90]. Yet, we found no differences in salivary free cortisol or salivary alpha-amylase between stress conditions. Previous research has demonstrated mixed findings with regards to the effects of threat of shock on HPA axis-related responses. One study found significant differences in cortisol responses between shock and safe conditions [26], whereas another study found differences between conditions only after dividing participants into “stress reactive” and “no stress” groups based on differing levels of physiological (cortisol levels) and self-reported stress (STAI scores) [25]. Taken together, we believe that our data indicates the consistent activation of early stress responses, specifically the noradrenergic sympathetic-adrenal-medullary (SAM) system, in the face of the threat of shock. However, there is no clear evidence of a reliable impact of shock on the relatively latent hypothalamic-pituitary-adrenal (HPA) axis, specifically glucocorticoid-related responses.

Furthermore, we did not observe changes in positive affect, negative affect or state anxiety in response to stress exposure. Rather, negative affect and anxiety both increased over time following both shock and vibrate conditions. Although statistically significant, these changes over time were relatively subtle (see Table 2). For instance, mean state anxiety scores fell within the “no or low anxiety” (20–37) classification range following both shock and vibrate conditions [84]. This is in contrast to previous research reporting significantly higher self-reported anxiety and negative affect under threat of shock [25, 45, 91–94]. Results indicate that the intensity of negative emotions induced by our stress procedure were relatively low, possibly contributing to the lack of HPA axis-related physiological differences between conditions.

In this study, participants received shocks following task errors. This was thought to emulate the anxiety experienced in response to anticipation of adverse consequences of error making in operational settings, like the return of hostile fire [33, 95]. Yet, it’s plausible that our shock delivery method lessened the perceived stress of the "threat-of-shock" by enhancing its predictability. Generally, stressors that can be anticipated tend to trigger lesser physiological stress reactions and are perceived as less distressing compared to unpredictable stressors [96]. For example, given that trials in the DMT task alternated between high and low levels of stimulus uncertainty, this likely led to varying ability among participants to predict whether shocks would be received. This variability in predictability of threat could have limited the extent to which stress was sustained across all trials, diminishing the overall effect of our stress manipulation.

Additionally, it is important to note that previous work showing emotional and physiological stress reactivity in response to threat of shock have sampled from populations of healthy civilians. Previous studies using threat of shock in military populations have not included measures of subjective emotional responses or HPA axis response. Instead, these studies have relied on heart rate or heart rate variability (HRV) as a physiological index of stress [17, 93, 97]. Sympathetic nervous system (SNS) activity pre, during and post-stressor is a critical component of the overall ANS stress response [98]. Crucially, although sympathetic activation has been linked to psychological stress and cognitive function, previously reported changes in HRV could also indicate that these threat of shock effects reflect non-specific arousal. Taken together, it remains unclear whether the threat of shock paradigm reliability induces stress in our population of interest, or whether previous results are merely due to general arousal. Future work should include both subjective and physiological measures to better assess the magnitude and specificity of this stress-induction technique for Soldiers.

### How does threat of shock influence memory, spatial orienting, and shoot/don’t shoot decisions?

Threat of shock did not influence object recognition or spatial orienting. This is inconsistent with previous empirical and meta-analytic findings suggesting that induced anxiety impairs explicit memory retrieval [99]. Here we found no influence of a TOS stress manipulation for safe-encoding/threat-retrieval, regardless of modality (spatial vs. object). Some have proposed that discrepancies in the timing of stress induction—whether during memory encoding or retrieval—might contribute to the inconsistent findings concerning the relationship between anxiety and memory [45, 46, 100]. We also found limited evidence of overall heightened stress during the TOS condition, making it challenging to interpret effects of TOS on spatial and object memory since stress conditions were mismatched across encoding and retrieval. However, recent work has shown that stress responsiveness may be another important factor driving effects of TOS on memory retrieval. In another reported effort using the DeMUS data set we showed that reduced stress responsiveness, as measured by lower salivary cortisol levels, was associated with higher discriminability to detect target objects on the BOLO list from lures in the RMT [101]. One methodological advantage of TOS when studying memory is that it allows stress to be independently manipulated across memory stages. Thus, additional research may help further elucidate the complexities of individual differences and stress exposure timing on anxiety-memory relations across memory modalities.

We also found no differences in shoot/don’t shoot discriminability (d’) between TOS conditions. These findings are inconsistent with previous work demonstrating a negative impact of shock on shoot/don’t shoot decisions [17, 33, 39]. It is important to note that previous work has compared shock to no-feedback conditions [33], making it challenging to compare the effects observed under shock and vibrate conditions examined here. Nonetheless, this body of work has shown that threat of shock leads to a more liberal decision criterion and increased false alarms on shoot/don’t shoot tasks, which we did not observe in our data.

As mentioned, one methodological factor that could have contributed to the discrepancy in results observed here is the predictability of threat. Studies employing predictable threat of shock as a relatively high-stakes outcome for decision errors (i.e., incorrectly orienting, missing a threat, or false-alarming to a non-threat) have demonstrated decrements in shoot/don’t shoot performance [17, 33, 39]. Interestingly, unpredictable threat of shock can lead to improvements on inhibitory control tasks, like a shoot don’t shoot task (i.e., go/no-go task). In these studies, participants are better able to withhold responses to infrequently presented no-go stimuli when under shock compared to safe conditions. Furthermore, this result has been replicated several times [31, 102, 103]. Given that our stimuli varied in level of uncertainty they also likely varied in subsequent predictability of threat. Thus, effects of predictable and unpredictable threat may have nullified any changes in shoot/don’t shoot performance that may have been observed under each threat type alone. Conversely, in real-life situations, there are rarely cues that can predict the emergence of a threat with absolute accuracy. Therefore, future work should compare how the relative predictability of threat impacts memory, spatial cognition or inhibitory control.

### How does threat of shock influence uncertain shoot/don’t shoot decisions?

Shoot/don’t shoot performance was influenced by uncertainty but did not differ between stress conditions. Consistent with previous work, we found that participants were slower, less accurate and less sensitive to the difference between friends (noise) and foes (signal) under conditions of low compared to high stimulus clarity (as demonstrated by lower *d’* scores) [72, 104]. Interestingly, further exploration demonstrated this difference in sensitivity was being driven by lower hit rates under conditions of low uncertainty rather than high uncertainty, as hypothesized. However, this result was likely driven by a shift in response bias where participants were more biased towards responding yes (i.e. shooting) in the high than low uncertainty condition. This shift in response strategy led to more correct identifications of enemy targets, but also more friendly engagements.

Although we did not observe differences in shoot/don’t shoot discriminability across our TOS conditions, we did find differences in decision distance (proxy to decreased response time) and confidence due to stress. Results indicated that participants responded to targets sooner but with lower confidence when under shock. Interestingly, participants were quicker to respond when the outcome of their decision would entail an aversive shock. In safe conditions, participants were slower to respond under conditions of high uncertainty, taking their time to identify and then fire. However, under threat of shock, participants fired at targets when they were farther away (responded faster) regardless of the clarity of the camouflage patterns. This is counter to the notion that threat may increase cautious behavior to promote harm avoidance. However, this did not appear to impact the accuracy of their shoot or don’t shoot decisions. Previous research has demonstrated mixed results with regards to effects of threat of shock on reaction time, or this measure is often unreported [17, 33, 39, 44], so interpretation of these initial results is limited.

We predicted that when faced with stress, it is likely adaptive to view potentially dangerous stimuli as threatening until contextual information proves otherwise (i.e., identify targets as enemies). However, it appears that when under conditions of high uncertainty (i.e., ambiguous discrimination between friends and foes) participants responded quicker and adopted a more liberal bias for identifying targets as threatening, regardless of stress condition. Research has shown that humans often use heuristics, or biases, to make decisions when certainty is low and ambiguity is high [105–107]. However, here, it appears Soldiers’ may be relying on certain cognitive biases to guide decisions irrespective of their current threat level. Indeed, during out-brief interviews, Patton and colleagues [33] noted that many participants in their study reported shooting at all targets because they felt they had a better chance of not being “shot”. Similarly, it appears that our sample may have adopted a maladaptive response tendency across safe and threatening contexts. It is possible that certain response patterns may emerge because of training or performing in high-stakes environments. However, decisions are not always binary (shoot or don’t shoot) in high stakes contexts. For instance, a Solider may need to prioritize multiple threats (e.g., based on distance from the shooter, or type of weapon) or decide between multiple use-of-force judgments (e.g. verbal responses, hard controls, and soft controls). Future work should also examine how certain responses biases may or may not impact other types of decisions pertinent to shoot/don’t shoot decisions for individuals high stress occupations.

### Limitations and future directions

In the present study, we found mixed evidence of successful stress manipulation, which is one reason why we did not observe more differences in DeMUS performance between our TOS stress conditions. To further understand the relationships between emotional stress, uncertainty and cognitive performance, future research should expand upon these findings in several ways.

First, several design differences may account for the inconsistent findings within the threat of shock literature. Threat of shock provides a well-controlled manipulation of stress and state anxiety in a within-subjects design but there is no “standard” paradigm [44]. For example, some studies manipulate threat between participants and some within; some utilize performance-contingent shocks and others administer unpredictable shocks which are unrelated to task performance. We chose performance-based shock because of its relevance to high stakes military contexts (i.e., failing to identify and/or eliminate a threat could have imminent life or death consequences). However, as Robinson and colleagues [45] have suggested, a clearer understanding of the effects of shock may be achievable if more methodological variables (e.g., frequency of shock delivery, block length) were held consistent across studies. Given that there are still very few studies utilizing the threat of shock paradigm, it is not surprising that there is much to be understood about how threat either does, or does not, induce cognitive changes. Additionally, future work may look to manipulate and explore not only the predictability of when threat (i.e., shock) will occur, but also the temporal proximity, likelihood of occurrence and control over shock exposure. Further examining how factors of uncertainty and controllability over perceived stressors influences decision making, and decision making in response to uncertainty, will help to generalize findings to real-world high-stakes contexts.

Second, the timing of our subjective and physiological sampling may have reduced our ability to detect any stress-induced changes. Due safety protocols for collecting human subject’s data and bio samples during the ongoing SARS-CoV-2 pandemic, we were limited in several ways. First, the additional time required to don personal protective equipment (e.g., masks, gloves, glasses, gowns, face shields), carefully handle biospecimen, sanitize hands, social distance, and sanitize lab spaces and equipment resulted in lengthy study sessions. To limit the time burden placed on both researchers and participants, all study sessions were scheduled between 7:00 and 11:00 am. However, prior research has established that a diurnal drop in cortisol level occurs throughout the day [90]. To control for diurnal rhythms in cortisol, study sessions are ideally scheduled between the hours of 1:00 and 6:00 pm [16]. With our sessions scheduled in the morning, a more robust cortisol response would’ve been required to counteract this natural drop. Since previous work using threat of shock in military populations has not included stress-specific biomarkers (e.g., glucocorticoid (cortisol) and/or proinflammatory cytokines) [16, 20, 108], the effective magnitude of this particular stressor remains unclear.

Additionally, although we assessed affect and state anxiety before and after completion of shock and vibrate sessions, these were administered roughly 5 minutes following the stress cessation. However, affect is transient, particularly when it is of low intensity, and can be diminished by engagement in distracting tasks [109–111]. Thus, the short walk from the virtual reality cave to an isolated testing room could have served to distract participants and diminish any negative affect or anxiety experienced. Thus, the timing of these assessments may have limited the sensitivity to detect effects of our stress manipulation. Despite this, previous research using other stress manipulation techniques (TSST) have demonstrated a relationship between cortisol responses and emotional responses [112]. It will be useful for future work to consider inclusion and timing of such measures to expand our understanding of the correspondence of HPA axis and emotional responses when using the threat of shock paradigm. This will also help to establish the efficacy and generalizability of this paradigm for inducing stress across various populations of interest.

Finally, an individuals’ trait characteristics can impact performance under stress. We recently demonstrated how various cognitive, health, physical, and social-emotional traits can imact performance on the DeMUS task (See [101]). However, it is possible that other traits, not examined here, may have both impacted the effectiveness of the threat of shock manipulation and subsequent performance. For example, intolerance of uncertainty, or an inability to tolerate the unpleasant response triggered by a lack of information, is a dispositional characteristic that can be a factor in the development and maintenance of stress and anxiety disorders [113, 114]. Individuals with high stress jobs, who train and operate in dangerous and uncertain environments, differ in their intolerance of uncertainty, as compared to a community or civilian sample [115]. Research shows that individual differences in intolerance of uncertainty can influence how people perceive and respond to potential threats, leading to varying anxiety levels even under similar conditions [116]. Thus, future work should explore the extent to this trait characteristic impacts stress responsiveness and cognitive performance in a threat of shock paradigm.

## Conclusions

In conclusion, in the current study threat of shock did not significantly alter higher order cognitive processes involved in recognition memory, spatial orienting or shoot/don’t shoot decisions. However, stress experienced in military contexts can be difficult to induce in laboratory settings. Although our hypotheses related to stress-induced changes to behavior were not supported, results highlight several important methodological nuances that should be considered when measuring and interpreting responses to ambiguous information in stressful contexts. Importantly, even slight shifts in cognitive processing may determine differences in perceiving information as safe vs. threatening, which could have significant behavioral consequences for military personnel. Thus, future research should continue to characterize decision-making in high-stakes environments.
